# Changes in the proteomes of the hemocytes and fat bodies of the flesh fly *Sarcophaga bullata *larvae after infection by *Escherichia coli*

**DOI:** 10.1186/1477-5956-8-1

**Published:** 2010-01-13

**Authors:** Alice Masova, Miloslav Sanda, Jiri Jiracek, Irena Selicharova

**Affiliations:** 1 Institute of Organic Chemistry and Biochemistry, Academy of Sciences of the Czech Republic, vvi Flemingovo nám 2, 166 10 Praha, Czech Republic

## Abstract

**Background:**

Insects have an efficient self-defense system that is based on innate immunity. Recent findings have disclosed many parallels between human and insect innate immunity, and simultaneously fine differences in the processes between various species have been revealed. Studies on the immune systems of various insect species may uncover the differences in their host defense strategies.

**Results:**

We analyzed the proteomes of the hemocytes and fat bodies of *Sarcophaga bullata *larvae after infection by *Escherichia coli*. The 2-DE gels of the hemocytes and fat bodies of infected larvae were compared with those of aseptically injured larvae. Our analysis included the construction of protein maps of the hemocyte cells and cells from fat bodies, the identification of the changed proteins, in response to infection, using LC-MS/MS, and the estimation of the trends in expression of these proteins at three time points (30 min, 6 hours and 22 hours) after infection. In total, seven changed spots were found in the hemocytes, and four changed spots were found in the fat bodies. Three types of trends in protein expression were observed. Cofilin and transgelin were undetectable at 30 min after infection but were continuously up-regulated in the induced larvae after 22 hours. A prophenoloxidase isoform and lectin subunit α were slightly up-regulated at 30 min after infection, and their protein levels reached the highest points after 6 hours but decreased after 22 hours. T-Complex subunit α, GST, ferritin-like protein and an anterior fat body protein (regucalcin homologue) were down-regulated at 22 hours after infection.

**Conclusions:**

Many proteins identified in our study corresponded to the proteins identified in other insects. Compared to the former studies performed in insects, we presented 2-D protein maps of the hemocytes and fat bodies and showed the trends in expression of the immune-elicited proteins.

## Background

Insects are a major group of arthropods and the most diverse group of animals on Earth. One of the reasons that insects prosper on Earth is their efficient self-defense system, which is based on innate immunity [1]. Innate immunity involves both cellular and humoral reactions, including phagocytosis, the activation of proteolytic cascades and the synthesis of potent antimicrobial peptides [2]. Many parallels between human and insect innate immunity have been disclosed, and simultaneously fine differences in the processes between various species have been revealed [2-4]. The most recent findings indicate that a primitive form of adaptive immunity exists in insects. Such conclusions are based on the observed diversification of immune receptor molecules in insects [5], the specificity of the outcome of infection depending on the interactions between host and pathogen [6] and the possibility of the priming of insect immune system by non-pathogenic bacteria [7].

Pilot studies on insect innate immunity were performed using the fruit fly, *Drosophila melanogaster*, as a model organism [2]. Further studies on the immune systems of other insect species, such as *Lepidoptera*, contributed to our understanding of the processes and uncovered the differences in their host defense strategies [8,9].

Flesh flies proved to be an important model for the investigations on diapause [10], cold hardiness [11] and they are particularly important in forensic studies [12]. Most flesh flies breed on decaying materials (carcasses and excrements). They survive in an environment full of parasites, bacteria and other infectious organisms. A powerful defense system should be anticipated in these species. Therefore, flesh flies is a useful model for studying innate immunity [13,14]. Here we presented a proteomic analysis of the immune-challenged larvae of *Sarcophaga (Neobellieria) bullata *(Parker 1916). This study followed our previous attempts to isolate and characterize new antimicrobial compounds from *S. bullata *larvae [15,16].

Proteomics can capture the changes on the protein level (e.g. post-translational processing) that are unapproachable by genomics or transcriptomics. Of course, proteomics must overcome many pitfalls in methodology and bioinformatics [17]. Proteins are extremely difficult to analyze due to the wide spectrum of their physicochemical characteristics as well as the naturally wide range of protein abundances. Nevertheless, the promises of proteomic research for the future are indisputable.

The central proteomic methodology combines two-dimensional electrophoresis (2-DE) with mass spectrometry (MS) for the identification of selected proteins. The methodology has been applied in several studies of the immune response in insects. Hemolymph, a body fluid of insects, which flows freely within the body cavity and plays many important biological roles, was analyzed in most cases. The pioneering studies were devoted to *Drosophila *[18-20]. Nevertheless, several other insect species, such as silkworm [21,22], mosquitoes (vectors for human parasites) [23], honey bee (a producer of honey and pollinator of plants) [24] or tobacco hornworm (a damaging pest of tobacco plants) [25], have been analyzed. The known immune-relevant proteins, such as several immune-recognition proteins, serine protease inhibitors and ferritins, together with some other proteins of different classes were found in these studies [18-24]. The specificity of the induced changes in respect to the infectious stimulus was highlighted [20]. Differences in the elicited immune responses depending on the period of sampling (minutes, hours or days post-infection) [19,23], the developmental stage of the animal (larvae versus adult) [24] and the type of the sample (hemolymph versus whole body) [23] were also evidenced.

We chose fat bodies and hemocytes for our proteomic analyses because they are responsible for the systemic immune response in insects. We found two studies that utilized these types of cells for proteomic analysis of the immune response. Loseva and Engstrom [26] analyzed the hemocytes derived from the *Drosophila *cell line mbn-2. They found 24 proteins changed after 30 min and 6 hours in response to lipopolysaccharide (*E. coli*). Wang et al. [22] studied lipopolysaccharide-induced proteins in the silkworm. They found 1 protein spot induced in fat body cells and 4 spots induced in hemolymph at 24 hours after lipopolysaccharide injection.

Considering the kinetics of the observed changes as an important aspect in expression profiling, we compared the 2-DE protein profiles of the hemocytes and fat bodies of *S. bullata *larvae at three time points (30 min, 6 hours and 22 hours). The immune challenge was elicited by injection of a bacterial suspension of *E. coli*. The control larvae were injured with a sterile entomological pin. We identified several proteins that were up- or down-regulated upon infection in *S. bullata *larvae and estimated the time course of their expression.

## Results

### Protein identification and construction of 2-D protein maps of the *S. bullata *larvae hemocytes and fat bodies

**Figure 1:**
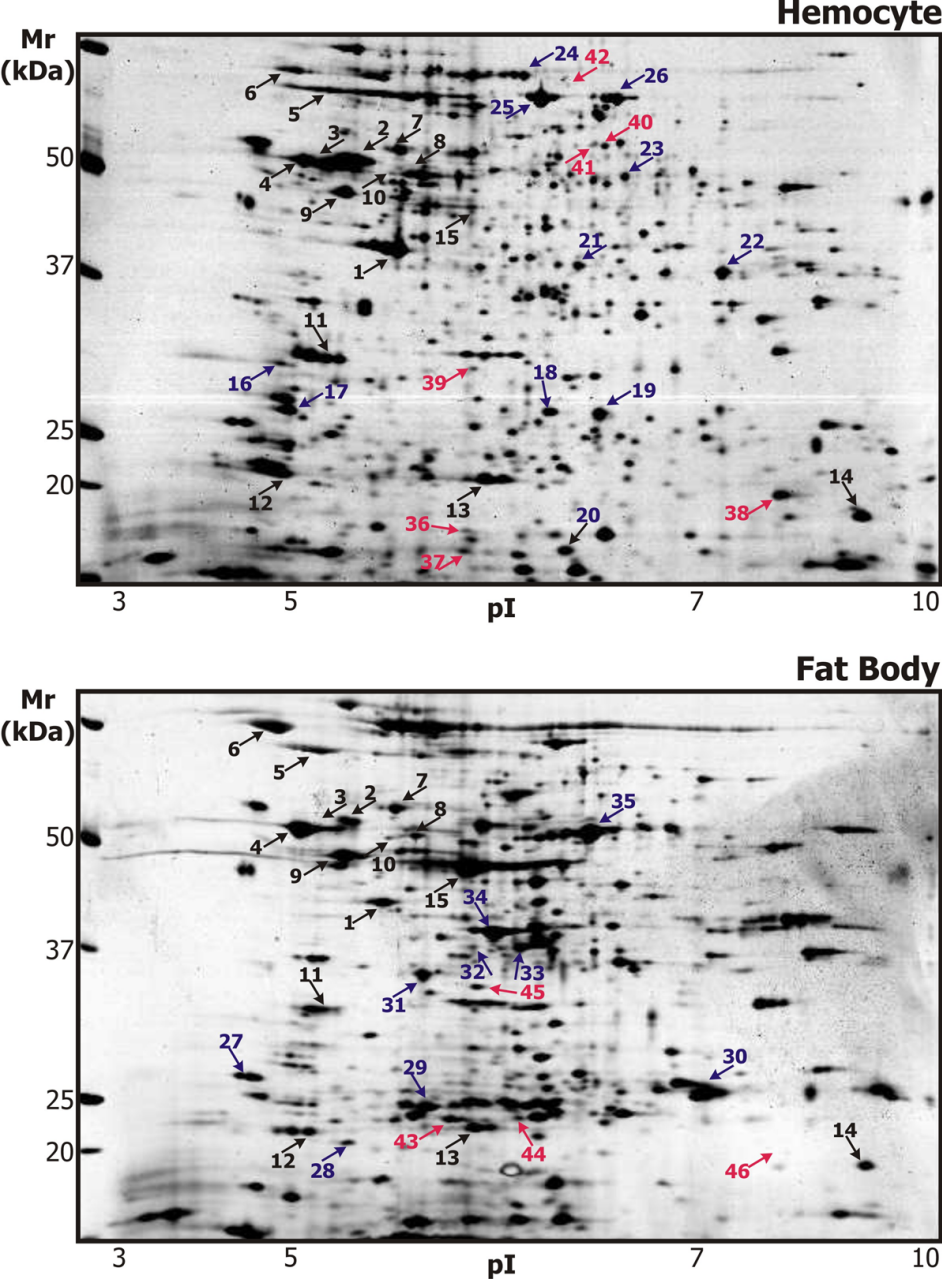
**Representative 2-DE maps of proteins of the *S. bullata *larvae hemocytes and fat bodies**. Solubilized proteins from the *S. Bullata *hemocytes and fat bodies, 6 hours after the injection (induced) or the treatment with sterile entomological pin (non-induced), were focused on IPG strips pH 3-10 NL and separated in SDS-polyacrylamide gradient gels (8-16%). The gels were silver stained. The proteins identified in numbered spots are listed in Additional file 1. The proteins identified in both 2-D protein maps are in black; proteins identified either in the hemocytes or in the fat bodies are in blue, and the proteins changed after immune challenge are in red.

The 2-D protein maps of the hemocytes and fat bodies were compared and the positions of common characteristic spots were assigned. Representative silver-stained 2-DE gels of the hemocytes and fat bodies of *S. bullata *larvae 6 hours after aseptic injury (non-induced) are shown in Figure 1. The characteristic spots are marked with the same numbers in both protein maps. All proteins that were identified from the 2-D protein maps of the *S. bullata *larvae hemocytes and fat bodies are described in Additional file 1.

The proteins in the preparative gels were identified by LC-MS/MS after digestion with trypsin. The genome of *S. bullata *has not been sequenced. Nevertheless, we succeeded in identification of many proteins by Sequest and Bioworks software searches against the UniProt protein database. Most proteins were identified with high probability by cross-matching with the *Drosophila *databases and a few proteins matched other insect databases, such as *Bombyx mori *or *Lucilia cuprina*. Identification of several proteins matched *Sarcophaga *entries.

Relative molecular weights (Mr) and isoelectric points (pI) were estimated from the positions of the spots in the gels. The measured pI values were only rough estimates because the nonlinear IPG strips in the pH 3-10 range were used. Nevertheless, the locations of the identified proteins in our gels in terms of Mr/pI were in good agreement with the theoretical Mr/pI of the proteins in the cross-matched databases, with a few exceptions that are discussed further. In the cases where multiple proteins were identified in the database search after MS/MS, we considered the identification as the one with the highest number of peptides assigned and the best correlation of the measured and theoretical Mr/pI values.

We succeeded in identifying 45 proteins. Although the 2-D protein maps from the hemocytes and fat bodies differed considerably, the main cellular proteins could be mutually assigned. The constituents of the cytoskeleton (actin, tubulins), the molecular chaperones (HSPs, protein disulfide isomerase) and the housekeeping enzymes (ATP synthase, GST) formed a characteristic picture that helped to match the 2-D protein maps. Some proteins were identified from either the hemocytes or fat bodies. Among them, a few more spots could be assigned between the 2-D protein maps (for example, spots no. 18, 27 and 28).

Several proteins that were characteristic or exclusive of each cell type were identified. Prophenoloxidases (spots no. 24, 25 and 26), together with fructose-bisphosphate aldolase (spot no. 22) and HSP 25 (spot no. 19), were characteristic for the hemocytes.

Three large spot clusters (no. 15, 34 and 35) were the main features in the 2-D protein map of the fat bodies. Storage protein-binding protein (hexamerin receptor) was identified in each of the spots. However, the theoretical Mr of this protein is much larger than the Mr of the observed spots. Because hexamerin receptors in dipteran insects are subjected to three post-translational cleavages [27], we think that the three spots correspond to the successive cleavage products. Other characteristic protein spots in the fat bodies were identified as arginine kinase (spot no. 33), glycine N-methyltransferase (spot no. 31) and development-specific 25 kDa protein (spot no. 30).

### Analysis of changes in the proteomes of the *S. bullata *larvae hemocytes and fat bodies after immune challenge

The hemocytes and fat bodies of induced and non-induced larvae were isolated in intervals of 30 min, 6 hours and 22 hours post-injection. The gels prepared from the samples from the different time points were mutually similar and did not differ dramatically.

We typically detected about 400 spots in the gels prepared from the hemocytes of *S. bullata *larvae and about 280 spots were matched to every gel in the match-sets created for each time point. The correlation coefficients between technical replicates of gels ranged from 0.7 to 0.9.

About 350 spots were detected in the gels prepared from the fat bodies and about 250 spots were matched to every gel in the match-sets created for each time point. The correlation coefficients between technical replicates of gels ranged from 0.6 to 0.8.

Altogether, six match-sets were created in PDQuest. The automatically assigned spots that changed at least twofold with 95% probability in each match-set were subjected to visual survey. Very faint spots, spots located in marginal areas of the gels or spots within streaks and spot clusters were excluded. The differentially expressed proteins that fulfilled our criteria of reliability in terms of appearance, location, fold change (at least two) and probability (95% in Student t-test) were subjected to identification and are described in the separate sections of the Additional file 1 (proteins changed in the hemocytes and proteins changed in the fat bodies). In total, seven changed spots were found in the hemocytes, and four changed spots were found in the fat bodies.

### The trends in expression of spots changed after immune challenge

**Figure 2:**
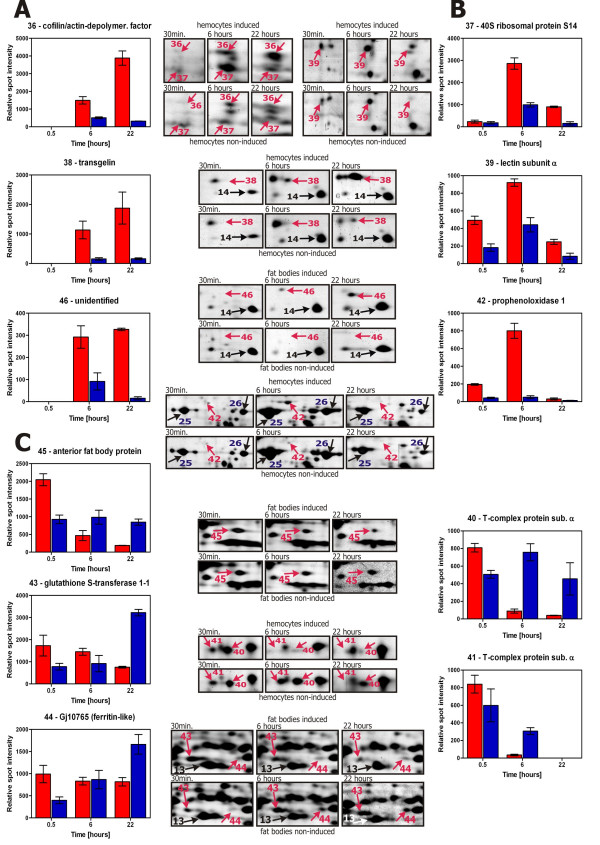
**Protein spots changed in the *S. bullata *larvae hemocytes and fat bodies after immune challenge**. Differentially expressed protein spots listed in the Additional file 2 are shown in the enlarged sections of representative gels from the *S. bullata *larvae hemocytes and fat bodies at three time points: 30 min, 6 hours and 22 hours post-injection. The relative intensities of particular spots including the standard error of the mean (n = 3) from the PDQuest output are shown in the bar graphs: red bars for induced larvae and blue bars for non-induced larvae. Panel A shows the spots continuously up-regulated in induced larvae. Panel B shows the spots up-regulated in induced larvae at 6 hours post-injection and then down-regulated. Panel C shows the spots down-regulated in induced larvae at 22 hours post-injection.

The spots that were considered reliable in one of the time points (30 min, 6 hours and 22 hours) were searched in the gels from the samples prepared from the other time points. We calculated the ratios (fold change) between the averaged spot quantities in the induced and non-induced hemocytes and fat bodies at each time point. The quantities were calculated by PDQuest after normalization on the total quantity of the valid spots in a given gel. To allow the comparison of the relative spot quantities between match-sets, the ratios of the quantities of several invariable spots between match-sets were calculated. The quantities of the spots of interest were adjusted with the calculated ratios (2:1:2 for the hemocytes and 1:1.5:1.2 for the fat bodies with respect to the time points of 30 min: 6 hours: 22 hours). The fold changes and estimated relative quantities of the spots are shown in Additional file 2. The graphical presentation of the data given in the Additional file 2, together with the representative sections of the gels showing the spots, is presented in Figure 2.

Basically, three types of trends in the spot expression were observed (Figure 2). Spots no. 36, 38 and 46 were not detectable at 30 min post-injection and were continuously up-regulated in the induced larvae after 22 hours (panel A in Figure 2). Spots no. 37, 39 and 42 were slightly up-regulated at 30 min post-injection, and their protein levels reached the highest points after 6 hours but decreased after 22 hours (panel B in Figure 2). Certain increase in the expression of these spots could be observed in the non-induced larvae, probably in reaction to the injury with the sterile pin. The trends in expression of spots no. 40, 41, 43, 44 and 45 were difficult to interpret. The spots appeared to be down-regulated at 22 hours post-injection, and their amounts mainly decreased with time in the induced larvae; however, in the non-induced larvae (after aseptic injury), the intensities of some of these spots increased, while, of the other spots, they decreased or remained unchanged (panel C in Figure 2).

### The proteins changed after immune challenge in the *S. bullata *hemocytes and fat bodies

We found seven significantly changed protein spots between the induced and non-induced hemocytes of *S. bullata *larvae.

Spot no. 36 was identified as cofilin/actin depolymerization factor. Similar to the result from Loseva and Engstrom [26], we detected two isoforms of this protein (spots no. 20 and 36). Only the more acidic form (probably post-translationally modified by phosphorylation) increased after immune challenge.

Spot no. 38 was identified as transgelin. It is an actin-interacting protein that cross-links actin filaments. A protein in the same location with the same trend in expression after immune challenge was also observed in fat bodies (spot no. 46).

Spot no. 39 was identified as lectin subunit α, an immune defense protein that is induced upon immune challenge in flesh flies [14]. Apparently, it was also induced by the sterile injury, but in lower extent compared to the induction by the bacterial infection (Figure 2, panel B).

Spot no. 42 was identified as prophenoloxidase 1. Interestingly, the NADH-ubiquinone oxidoreductase subunit of the mitochondrial membrane respiratory chain was identified in the same spot from the non-induced hemocytes. Prophenoloxidase 1 was also identified in two large neighboring spots (no. 25 and 26), but only spot no. 42 was selectively induced upon bacterial infection.

Spot no. 37 was repeatedly (in four experiments) identified as 40S ribosomal protein S14. However, the theoretical pI (10.3) of this protein differed considerably from the estimated pI (6) of the spot; therefore, we did not consider this identification reliable.

T-complex protein 1 subunit α is a component of the hetero-oligomeric chaperonin complex that assists protein folding upon ATP hydrolysis [28]. The protein was detected in two neighboring spots (no. 40 and 41). Both spots were down-regulated in the induced *S. bullata *larvae hemocytes.

Four significantly changed proteins spots were found between the induced and non-induced fat bodies of *S. bullata *larvae. We have already commented on the expression of spot no. 46.

Spot no. 45 was identified as an anterior fat body protein. The spot was about twofold up-regulated after 30 min and then decreased in the induced fat bodies, while its amount remained constant in the non-induced larvae.

The intensities of spots no. 43 and 44 increased with time in the non-induced larvae. Spot no. 43 and its neighboring spot no. 13 were both identified as GST. We speculate that spot no. 43 might be post-translationally modified. The similar trend in expression was observed for spot no. 44, which was identified as ferritin-like protein. Another type of ferritin-like protein was identified in spot no. 29.

## Discussion

We performed a proteomic analysis of the *S. bullata *larvae hemocytes and fat bodies after infection by *E. coli*. The protein preparations of the infected larvae were compared with those of the aseptically injured larvae. Most of the proteomic studies on insect immunity compared the hemolymph protein profiles between the control and immune-challenged animals [19,20,24], while the proteome profiling of the hemocytes or fat bodies were reported only in a few cases [22,26].

### The protein map of the *S. bullata *hemocytes

Hemocytes are immune surveillance cells in insects. In *Drosophila*, there are three types of hemocytes (lamellocytes, plasmatocytes and crystal cells) with distinct functions. The protein map of the *Drosophila *blood cell-derived cell line, mbn-2, was established by Loseva and Engstrom [26]. They identified 65 spots by MALDI-TOF. The localization and identities of many spots were comparable to our 2-D map of the *Sarcophaga *hemocytes. The main distinguishing feature of our map was the presence of two large spots no. 25 and 26 identified as prophenoloxidase 1, which are not apparent in the protein map of the mbn-2 cell line. These spots probably originated from a fraction of the hemocytes in *Sarcophaga*, which synthesize prophenoloxidase and are analogues to *Drosophila *crystal cells. The intensities of these spots seemed to change in the time course according to our analysis (see Figure 2, spots no. 25 and 26), suggesting that there probably were changes in the proportions of the hemocyte types.

### Changes in the proteomes of the *S. bullata *larvae hemocytes after immune challenge Prophenoloxidase

Activation of prophenoloxidases and subsequent melanization are in the front line of host defense in insects [4,29]. Activation of prophenoloxidases is a carefully regulated process [30]. Several prophenoloxidase genes exist in different arthropods. Some studies suggest that the different prophenoloxidases may have different functions [31]. In *Drosophila*, prophenoloxidases 1 and 2 were expressed constitutively but prophenoloxidase 3 was upregulated after infection by parasitoid wasps [32]. The constitutive expression of prophenoloxidases was evidenced by many studies [8,24]. Recently, we studied the time-dependent immune response of eight *S. bullata *genes in the larvae using real-time PCR. We did not detect any change in expression of the genes that encode prophenoloxidase 1 and 2 [16].

Li et al. [33] analyzed prophenoloxidases in *Aedes aegypti *larvae, which have nine prophenoloxidase genes. They indicated that the proteolytic processing of prophenoloxidases after their synthesis might occur in *A. aegypti*. We identified *Sarcophaga *prophenoloxidase 1 in three spots (no. 25, 26 and 42). The spots corresponding to prophenoloxidase 1 had a slightly lower Mr than the theoretical value, suggesting that they might be proteolytically processed, similarly to the *A. eagypti *prophenoloxidases. Moreover, the three spots had different pIs. We detected an isoform (spot no. 42, Figure 2) that was specifically and selectively induced in response to infection. Whether this isoform results from post-translational processing or whether more prophenoloxidase genes exist in *S. bullata *is not clear. All of these data imply that not only is the prophenoloxidase activation cascade a complicated and tightly regulated process, but certain regulation might also exist at the level of post-translational processing of the precursor and/or differential expression of different genes.

### Lectin subunit α

Another immune-related protein induced in the hemocytes was lectin subunit α C-type lectins are calcium-dependent carbohydrate-binding proteins that can bind to the terminal sugars on the surface of microorganisms. Inducible lectins have been found in flesh flies [14] and other insects, such as silkworm [34]. No lectins are known to be induced upon infection in *Drosophila*, although C-type lectins are encoded in the genome [35]. Tanji et al. [14] studied the regulation of expression of a *Sarcophaga *lectin gene. The induction of the lectin was not infection-specific. It was also triggered by aseptic injury. Nevertheless, the expression was higher in response to the infectious stimulus compared to the aseptic injury. The gene was transcribed mainly in the fat body. We observed a trend in expression of the lectin that precisely corresponded to Tanji's findings (spot no. 39, Figure 2), except that we detected the protein in the hemocytes, not in the fat body.

### Other changed proteins in the *S. bullata *hemocytes

Cytoskeletal proteins and their regulators are implicated to perform various functions in immune cells as documented by many studies [19,26]. We found two cytoskeleton regulatory proteins that were up-regulated in response to the immune challenge in *S. bullata*: the acidic isoform of cofilin/actin depolymerization factor (spot no. 36) and transgelin (spot no. 38). Both proteins might facilitate cytoskeleton reorganization during phagocytosis.

The chaperonin T-complex has an indispensable role in the folding of cytoskeletal proteins [28]. T-complex protein 1 subunit α was found in two spots (spots no. 40 and 41, Figure 2), one of which probably corresponded to a phosphorylated form. Both spots were down-regulated in the immune-challenged larvae hemocytes. Two subunits (β and ε) of the chaperonin T-complex were up-regulated in the mbn-2 cell line [26]. At this point, it is not clear what the proper interpretation of these observations should be.

Spot no. 37 has been identified as 40S ribosomal protein S14. However, we do not consider this identification reliable (Table 1). Interestingly, two ribosomal proteins (marked I and J) were also increased in the proteome of *A. aegypti *larvae after infection by a parasite [23]. The measured pIs of the spots were 7.2 and 5.6, while the theoretical values were 9.8 and 11.4, respectively, according to ExPASy http://www.expasy.org/.

### The protein map of the *S. bullata *fat bodies

The insect fat body is an organ analogous to vertebrate adipose tissue and liver. It functions as a major organ for nutrient storage and energy metabolism [36]. We did not find any report presenting a 2-D protein map of the insect fat body. Shih and Fallon [37] performed 2-DE analysis of the mosquito fat body during the gonadotropic cycle, but they did not identify any proteins. Zao et al. [38] identified the differentially expressed proteins during larval molting of *Helicoverpa armigera*. While they also analyzed the fat body fraction, they did not give a description of the protein map. Zhou et al. [39] analyzed the changes in the proteomes of silkworms with various rearing diets and identified the proteins changed in the fat bodies, some of which corresponded to the proteins identified in our analysis.

Our 2-D protein map of the *S. bullata *fat body (Figure 1) provided the first detailed insight into the proteome of this important insect tissue. Beside the cytoskeletal and housekeeping proteins, we also identified the proteins that confirmed the roles of the fat body. Large amounts of storage protein-binding protein (hexamerin receptor) is related to the nutrient storage function of the fat body [27]. The importance of this organ for energy metabolism is demonstrated by the large amount of arginine kinase, which catalyzes the reversible trans-phosphorylation between phosphoarginine and ATP, thus buffering cellular ATP levels [40]. Its resemblance to the mammalian liver is demonstrated by the high amount of glycine N-methyltransferase, which is a characteristic enzyme of the liver and plays a regulatory role in the tissue concentrations of the essential methyl donor S-adenosylmethionine [41].

### Changes in the proteome of the *S. bullata *larvae fat bodies after immune challenge

The synthesis of antimicrobial peptides and other immune compounds takes place in the fat body [2]. The changes of the fat body proteome, triggered by the elicited immune responses, have not been systematically studied so far, except for one report from Wang et al. [22], who analyzed the fat body of *Bombyx mori *at 24 hours post-infection by *E. coli *lipopolysaccharides. They found only one spot, identified as anti-trypsin that was significantly changed in the fat body.

Our analysis provided the rather complicated data. Due to the large spot clusters that were characteristic of the fat body 2-D protein map, we were unable to properly match certain spots. Therefore, the possible changes in these spots could escape our analysis. Nevertheless, no dramatic changes in the fat body proteome were seen in the course of our analysis. Apparently, the immune challenge does not evoke dramatic changes in the proteomes of either the hemocytes or the fat bodies of *S. bullata *compared to the rather extensive alterations in the hemolymph proteomes or the whole body proteomes observed in other insects [20,23].

We found four significantly changed spots. Spot no. 46 was not identified. The other spots were identified as an anterior fat body protein (regucalcin homologue), GST and ferritin-like protein. All these proteins were reported to be differentially expressed in proteomic studies analyzing the hemolymph of immune-challenged insects [19,20,23]. The function of the anterior fat body protein, which is similar to mammalian liver regucalcin, has not been characterized in insects so far [42]. GST is a detoxifying enzyme, responsible for the biological neutralization of xenobiotics. Ferritin is an iron storage protein, important for iron homeostasis and it is also an acute-phase protein [43].

The proteins showed interesting trends in expression. They seemed to be up-regulated after 30 min but down-regulated after 22 hours in the infected fat body. We speculate that the proteins may be induced or post-translationally modified in the fat body and then secreted to the hemolymph in a differential manner that depends on the type of the immune challenge (injury versus infection). In this sense, elucidation of the regulation of the differential manners in expression and secretion of these immune relevant proteins will be the important next-step for the following study.

## Conclusions

To conclude, here we presented an extensive study of the immune response in flesh fly larvae to bacterial infection. Many proteins identified in our study corresponded to the findings of others, which pointed out the similarity of immune responses in different insect species. Compared to former studies performed in insects, we showed the trends in expression of immune-elicited proteins. The 2-D protein maps of the hemocytes and fat bodies were presented. Our results suggested that a considerable part of the immune response is managed through the post-translational modification of proteins, highlighting the usefulness of the proteomic approach. The extensive studies of insect immunity may contribute to our understanding of immune responses in humans.

## Methods

### Animals

*Sarcophaga (Neobellieria) bullata *(Parker 1916) larvae were reared at 200-300 specimens per batch on beef liver in disposable aluminum packets at 25°C. The third-instar larvae in their wandering period were collected.

*Escherichia coli *DBM 3001 were cultivated in Luria-Bertani broth at 37°C to the mid-exponential phase of growth, with a final concentration of cells at 10^9 ^cfu.ml^-1^. The cells were centrifuged at 3000 g for 10 min and washed twice with 0.1 M phosphate buffer, pH 7. Bacterial suspensions in physiological saline with the absorbance readings of 1.5 -1.8 at 550 nm (about 2 × 10^6 ^cells in 1 μl) were used for induction of *S. bullata *larvae.

### Preparation of protein samples

Larvae were immobilized by chilling on ice. The bacterial suspension (1 μl containing 2 × 10^6 ^cells) was injected into their abdomen using a calibrated glass capillary (induced). Control larvae were pricked with a sterile entomological pin (non-induced). The hemolymph was collected from 20 specimens of induced and non-induced larvae at 30 min, 6 hours and 22 hours post-injection. The immobilized larvae were pricked and gently squeezed to collect the hemolymph. The hemocytes were separated by centrifugation at 1000 g for 10 min at 4°C and washed twice with 50 mM Hepes buffer, supplemented with 90 mM NaCl, pH 7.4.

The fat bodies were isolated at 30 min, 6 hours and 22 hours post-injection from another batch of induced and non-induced larvae. Five larvae per treatment were anesthetized with CO_2_. The anterior and posterior tips were cut off with fine scissors, and the fat body was excised under a binocular microscope. The Malpighian tubules and tracheas were removed.

Both the fat bodies and hemocytes were homogenized with a Teflon pestle in 200 μl of lysis buffer (7 M urea, 2 M thiourea, 4% (w/v) CHAPS, 40 mM Tris, 65 mM DTT, 2% (v/v) ampholytes, pH 9-11), sonicated three times for 2 seconds and incubated for 30 min at room temperature. The cell debris was removed by centrifugation at 16000 g for 10 min, and the protein content was determined using the Bradford assay [44]. Samples were aliquoted and stored at -70°C.

### 2-DE

The cell lysate of hemocytes (70 μg of protein for analytical gels and 400 μg of protein for preparative gels) and the cell lysate of fat bodies (55 μg of protein for analytical gels and 315 μg of protein for preparative gels) in rehydration buffer (7 M urea, 2 M thiourea, 4% CHAPS, 50 mM DTT, 0.8% ampholytes, pH 3-10) were applied to 18 cm nonlinear IPG strips (pH 3-10; GE Healthcare, Uppsala, Sweden). 2-DE was performed on PROTEAN IEF cell and PROTEAN II XL systems (Bio-Rad, Hercules, USA). Active rehydration was carried for 12 hours at 50 V. IEF was performed with the stepwise voltage increases as follows: 250 V for 1 h, 500 V for 1 h, 1000 V for 2 h and 10,000 V for the time period necessary to reach 40,000 Vh. The focused strips were equilibrated for 30 min in a solution containing 6 M urea, 20% (v/v) glycerol, 2% (w/v) SDS, 0.05 M Tris-HCl, pH 8.8 and 2% (w/v) DTT with a trace of Bromophenol Blue. Afterwards, free thiol groups were alkylated for 30 min in the same solution containing 2.5% (w/v) iodoacetamide instead of DTT. SDS-PAGE on gradient gels (8-16%, 4% stacking gel, 19 × 22 cm) was performed in 0.025 M Tris, 0.192 M glycine and 0.1% (w/v) SDS running buffer for 1 h at 16 mA and about 9 h at 24 mA per gel until the Bromophenol Blue dye front reached the bottom of the gel. The analytical gels were stained with silver stain according to the Bloom method modified by Rabilloud [45]. Four analytical silver-stained gels in the nonlinear pH range of 3 to 10 were prepared from each sample. The preparative gels were stained with colloidal Coomassie stain [46]. All common chemicals were from Sigma (St. Louis, USA) and Fluka (Buchs, Switzerland).

### Image analysis

Gels were scanned with a GS-800 Calibrated Densitometer (Bio-Rad, Hercules, USA) at 400 dpi resolution. The images were further processed by PDQuest Advanced 8.0.1 2D Gel Analysis Software (Bio-Rad, Hercules, USA). The images were cropped to frame the same clusters of spots, which covered the area of the gels corresponding to Mr of 15 to 90 kDa and pI of 4.7 to 9.7. Three representative gels per each larvae population (the non-induced and the induced) were used to create a match-set. Individual match-sets were created for each period of time (30 min, 6 hours and 22 hours) and each type of samples (hemocytes and fat bodies). Together, six match-sets were created. The spot detection and matching were edited manually. The spot boundary tool was applied to detect large spots. The total density in valid spots was chosen as the normalization method. The differentially expressed proteins were searched based on the greater than twofold changes between the average expressions in groups of three representative replicate gels at the probability level p ≤ 0.05, determined by the Student's t-test. The spots detected as differentially expressed at each time point were searched in the corresponding match-sets (hemocytes or fat bodies) of the other time points, and their average quantities were calculated. The trends in the expression of the spots were estimated.

### Mass spectrometry and protein identification

The spots obtained from the PDQuest analyses with 95% probability (Student's t-test) and a more than twofold difference between the induced and non-induced larvae were considered for identification. We also identified selected characteristic spots, in the protein maps of the hemocytes and fat bodies of *S. bullata*. The relative molecular masses (Mr) and isoelectric points (pI) were estimated for each protein from its position in the gel. Proteins were excised from the preparative gels and were in-gel digested with trypsin [47]. The extracted peptides were concentrated in a SpeedVac (Thermo Fisher Scientific, USA). The resulting peptide mixture was analyzed by LC-MS/MS on an LTQ-ORBITRAP mass spectrometer (Thermo Fisher Scientific, Germany) coupled with a Rheos 2000 2-D capillary chromatography (Flux Instruments, Switzerland). The first dimension column was a Symetry C18, 180 μm × 20 mm × 3 μm (Waters, UK), and the second dimension column was a PepMap C18, 75 μm × 150 mm × 3 μm (LC Packings, USA). A data-dependent scan composed of one full MS scan (the resolution of 60,000) and three CID MS/MS scans (the resolution of 7,500) was used as the mass spectrometry method. The mass tolerance for peptide identification was 10 ppm. The mass tolerance for searching for fragment ions was 50 ppm. The identity of all peptides was confirmed by at least three fragment ions. The sequences were searched against the UniProt protein database (version accessed at November 1, 2008) by the Bioworks Browser 3.3.1 SP1 and Sequest 2.0 software (Thermo Fisher Scientific, USA) without restriction on the taxonomy.

## Competing interests

The authors declare that they have no competing interests.

## Authors' contributions

**AM **optimized the methodology for the preparation of *S. bullata *protein samples, prepared the 2-DE gels, prepared the samples for protein identification and contributed to the writing of the manuscript. **MS **performed the LC-MS/MS experiments and protein identifications. **JJ **contributed to the study concept and design, coordinated the study and critically revised the manuscript. **IS **designed and performed the analysis of 2-DE gels, prepared the samples for protein identification, performed the analysis, evaluation and interpretation of the data and drafted the manuscript. All authors read and approved the final manuscript.

## Supplementary Material

Additional file 1 **Proteins identified from the 2-DE gels of *S. bullata *larvae hemocytes and fat bodies**. The table is in the Microsoft word format (Table 1.doc). For each protein spot marked in Figure 1 we show the accession number to the Swiss-Prot database of the identified protein, the protein name or its abbreviation, the organism from which the protein identity was cross-matched, the theoretical and experimentally found relative molecular weight (in kDa) and isoelectric point (Mr/pI), the probability (P) that the identification by MS of a given protein is found by chance and the number of assigned and sequenced peptides (pept.). The protein map from which the identification was achieved is marked as H for hemocyte and FB for fat body. Click here for file

Additional file 2 **Trends in expression of proteins changed after immune challenge in *S. bullata *larvae hemocytes and fat bodies**. The table is in the Microsoft word format (Table 2.doc). Spots are numbered the same as in Additional file 1 and Figure 1. For each spot we show the accession number to the Swiss-Prot database, the protein name, estimated relative quantity including standard error of the mean (n = 3) in induced/non-induced larvae (I/N) from the PDQuest output and the fold change at three points of time; 30 min., 6 hours and 22 hours post-injection. Click here for file
