# Supplementary material for: Changes in the proteomes of the hemocytes and fat bodies of the flesh fly Sarcophaga bullata larvae after infection by Escherichia coli
Source: Proteome Sci. 2010 Jan 13;8:1. doi: 10.1186/1477-5956-8-1 (PMC2817646; doi:10.1186/1477-5956-8-1)
Supplement: Additional file 1 — Proteins identified from the 2-DE gels of S. bullata larvae hemocytes and fat bodies. The table is in the Microsoft word format (Table 1.doc). For each protein spot marked in Figure 1 we show the accession number to the Swiss-Prot database of the identified protein, the protein name or its abbreviation, the organism from which the protein identity was cross-matched, the theoretical and experimentally found relative molecular weight (in kDa) and isoelectric point (Mr/pI), the probability (P) that the identification by MS of a given protein is found by chance and the number of assigned and sequenced peptides (pept.). The protein map from which the identification was achieved is marked as H for hemocyte and FB for fat body. [file 1477-5956-8-1-S1.DOC]

**Table 1**

**Proteins identified from the 2-DE gels of *S. bullata* larvae hemocytes and fat bodies.**

| Spot no. | Access. no. | Protein name (function) | Organism | Meas.  Mr/pI | theor.  Mr/pI | P  in H | P  in FB | pept.  in H | pept.  in FB |
| --- | --- | --- | --- | --- | --- | --- | --- | --- | --- |
| **Proteins common to hemocytes and fat bodies that were identified in both protein maps** | | | | | | | | | |
| 1 | P10987 | Actin C5 | DROME | 42/5.4 | 41.5/5.5 | 1.8E-6 | 5.3E-10 | 8 | 14 |
| 2 | P06603 | Tubulin α-1 | DROME | 52/5.2 | 50/5.1 | 7.7E-11 | 4.2E-13 | 17 | 33 |
| 3 | Q24560 | Tubulin β-1 | DROME | 50/4.9 | 50/4.9 | 9.7E-9 | 7.5E-7 | 13 | 7 |
| 4 | P54399 | Protein disulfide-isomerase | DROME | 50/4.7 | 54/4.7 | 7.6E-8 | 5.8E-8 | 11 | 10 |
| 5 | P11147 | HSP 70 | DROME | 70/5 | 71/5.5 | 1.3E-6 | 1.6E-10 | 10 | 10 |
| 6 | P29844 | GRP 78 | DROME | 75/4.8 | 70/5.3 | 1.2E-10 | 4.1E-11 | 16 | 8 |
| 7 | O02649 | HSP 60 | DROME | 56/5.4 | 55/5 | 9.8E-6 | 3.6E-11 | 8 | 16 |
| 8 | B3NSB4 | GG22623 (Protein disulfide-isomerase) | DROER | 50/5.5 | 55/5.5 | 5.7E-8 | 7.1E-8 | 11 | 7 |
| 9 | Q05825 | ATP synthase sub. β, mitochondrial | DROME | 48/5 | 50/5 | 4.4E-10 | 3.6E-12 | 10 | 19 |
| 10 | P31409 | V-ATPase sub. B | DROME | 50/5.3 | 54/5.4 | 4.6E-11 | 2.6E-11 | 3 | 16 |
| 11 | P22464 | Annexin B9 | DROME | 32/5 | 36/4.9 | 7.0E-8 | 5.6E-4 | 17 | 6 |
| 12 | Q6XIN1 | TCTP | DROYA | 20/4.8 | 19/4.8 | 6.5E-10 | 9.8E-4 | 8 | 2 |
| 13 | P42860 | **Glutathione S-transferase 1-1** | LUCCU | 22/6 | 23/6.8 | 1.2E-8 | 4.4E-6 | 10 | 6 |
| 14 | B4KTE5 | **Peptidyl-prolyl cis-trans isomerase** | DROMO | 19/9 | 22/8.6 | 6.7E-7 | 5.0E-5 | 7 | 1 |
| ^a^15 | Q26654  O61491 | Storage protein-binding protein  **Flotillin-1** | SARPE  DROME | 47/6 | 133/6  47/5.6 | 1.9E-8  1.7E-7 | 8.5E-9 | 11  15 | 9 |
| **Proteins identified in the hemocytes** | | | | | | | | | |
| 16 | Q26636 | Cathepsin L heavy chain | SARPE | 29/4.7 | 19/4.8 | 1.0E-9 |  | 4 |  |
| 17 | P92177 | 14-3-3 protein ε | DROME | 27/4.8 | 29/4.8 | 8.6E-8 |  | 7 |  |
| 18 | Q94920 | **Porin** | DROME | 27/6.6 | 30/6.5 | 1.0E-4 |  | 4 |  |
| 19 | A1E385 | small HSP 25 | SARCR | 27/7 | 25/7.3 | 1.1E-8 |  | 6 |  |
| 20 | P45594 | **Cofilin/actin-depolymerizing factor** | DROME | 16/6.8 | 17/6.8 | 1.6E-8 |  | 15 |  |
| 21 | A4V2S3 | C-terminal binding protein | DROME | 40/6.8 | 42/6.5 | 3.9E-6 |  | 7 |  |
| 22 | P07764 | **Fructose-bisphosphate aldolase** | DROME | 40/7.6 | 39/7.4 | 4.0E-8 |  | 5 |  |
| 23 | P54385 | **Glutamate dehydrogenase, mitochondrial** | DROME | 50/6.7 | 56/7.4 | 6.4E-8 |  | 26 |  |
| 24 | Q9Y0B3 | **Prophenoloxidase 2** | SARBU | 75/6.5 | 80/6.1 | 2.5E-5 |  | 8 |  |
| 25 | Q9Y0B4 | **Prophenoloxidase 1** | SARBU | 70/6.6 | 79/6.1 | 1.8E-12 |  | 68 |  |
| 26 | Q9Y0B4 | **Prophenoloxidase 1** | SARBU | 70/7 | 79/6.1 | 9.0E-13 |  | 45 |  |
| **Proteins identified in the fat bodies** | | | | | | | | | |
| 27 | O96827 | Probable elongation factor 1-β | DROME | 26/4.6 | 24/4.4 |  | 3.8E-7 |  | 4 |
| 28 | B0WSM6 | **Lactoylglutathione lyase** | CULQU | 19/5.3 | 24/5.9 |  | 6.3E-4 |  | 2 |
| 29 | B4K9S1 | **GI24318 (ferritin-like)** | DROMO | 23/5.8 | 22/5.7 |  | 2.8E-4 |  | 4 |
| 30 | P23170 | **Development-specific 25 kDa protein** | SARPE | 25/7.6 | 28/7.6 |  | 1.9E-6 |  | 27 |
| 31 | B4K763 | **GI10501** ([glycine N-methyltransferase)](http://www.ebi.ac.uk/ego/DisplayGoTerm?id=GO:0017174) | DROMO | 35/5.8 | 33/5.8 |  | 9.7E-8 |  | 6 |
| 32 | B4N4V3 | **GK20439 (**[acyl-CoA dehydrogenase)](http://www.uniprot.org/uniprot/?query=family:"acyl-CoA+dehydrogenase+family") | DROWI | 37/6 | 46/6 |  | 2.4E-10 |  | 5 |
| 33 | P48610 | Arginine kinase | DROME | 37/6.5 | 39/6 |  | 6.6E-7 |  | 17 |
| 34 | Q26654 | Storage protein-binding protein | SARPE | 40/6.2 | 133/6 |  | 2.6e-8 |  | 2 |
| 35 | Q26654 | Storage protein-binding protein | SARPE | 50/6.8 | 133/6 |  | 7.9E-9 |  | 6 |
| **Proteins changed in hemocytes after immune challenge** | | | | | | | | | |
| 36 | P45594 | **Cofilin/actin-depolymerizing factor** | DROME | 17/6 | 17/6.8 | 5.4E-8 |  | 11 |  |
| ^b^37 | P14130 | **40S ribosomal protein S14** | DROME | 16/6 | 16/10.3 | 4.4E-6 |  | 4 |  |
| 38 | Q1HQ70 | Transgelin | BOMMO | 20/8.2 | 21/8.4 | 1.7E-7 |  | 6 |  |
| 39 | P05047 | **Lectin subunit α** | SARPE | 30/6 | 30/5.9 | 1.0E-11 |  | 9 |  |
| 40 | P12613 | **T-complex protein 1 sub. α** | DROME | 57/6.8 | 59/6.4 | 6.5E-8 |  | 22 |  |
| 41 | P12613 | **T-complex protein 1 sub. α** | DROME | 57/6.7 | 59/6.4 | 1.1E-6 |  | 11 |  |
| ^c^42 | Q9Y0B4  Q94511 | **Prophenoloxidase 1**  **NADH-ubiquinone oxidoreductase 75 kDa sub.** | SARBU  DROME | 73/6.7 | 79/6.1  76/5.9 | 5.0E-7  1.0E-5 |  | 14  2 |  |
| **Proteins changed in fat bodies after immune challenge** | | | | | | | | | |
| 43 | P42860 | **Glutathione S-transferase 1-1** | LUCCU | 22/5.9 | 23/6.8 |  | 3.9E-6 |  | 4 |
| 44 | B4M6F3 | **GJ10765 (ferritin-like)** | DROVI | 23/6.5 | 25/6.2 |  | 4.4E-6 |  | 4 |
| 45 | Q9NDP1 | **Anterior fat body protein** | SARPE | 34/6.1 | 34/5.9 |  | 3.9E-7 |  | 12 |
| ^d^46 |  | unidentified |  | 20/8.2 |  |  |  |  |  |

For each protein spot we show the accession number to the Swiss-Prot database of the identified protein, the protein name or its abbreviation, the organism from which the protein identity was cross-matched, **the** theoretical and experimentally found relative molecular weight (in kDa) and isoelectric point (Mr/pI), the probability (P) that the identification by MS of a given protein is found by chance and the number of assigned and sequenced peptides (pept.). The abbreviations of the organism names are as follows: **BOMMO -** [*Bombyx mori*,](http://www.uniprot.org/taxonomy/7091) **CULQU -** [*Culex quinquefasciatus*,](http://www.uniprot.org/taxonomy/7176)  **DROER -** [*Drosophila erecta*,](http://www.uniprot.org/taxonomy/7220)  **DROME -** [*Drosophila melanogaster*,](http://www.uniprot.org/taxonomy/7227)  **DROMO -** [*Drosophila mojavensis*,](http://www.uniprot.org/taxonomy/7230)  **DROVI -** [*Drosophila virilis*,](http://www.uniprot.org/taxonomy/7244)  **DROWI -** [*Drosophila willistoni*,](http://www.uniprot.org/taxonomy/7260)  **DROYA -** [*Drosophila yakuba*,](http://www.uniprot.org/taxonomy/7245)  **LUCCU -** [*Lucilia cuprina*,](http://www.uniprot.org/taxonomy/7375) **SARBU -** [*Sarcophaga bullata*,](http://www.uniprot.org/taxonomy/7385)  **SARCR -** [*Sarcophaga crassipalpis*](http://www.uniprot.org/taxonomy/59312)**, SARPE -** [*Sarcophaga peregrina*.](http://www.uniprot.org/taxonomy/7386) The protein map from which the identification was achieved is marked as H for hemocyte and FB for fat body. The spots are shown in Figure 1.

**a) The spot was highly abundant in the fat bodies in contrast to the hemocytes. Two proteins were identified with similar probability in the spot from the hemocytes.**

**b) Measured and theoretical values of pI differed inadequately. The identification was not reliable.**

**c) Prophenoloxidase was identified in the spot from induced hemocytes. NADH-ubiquinone oxidoreductase was identified from non-induced hemocytes.**

**d) The spot has the same localization in the 2-D protein map of the fat bodies as the spot 38 (transgelin) identified from the hemocytes.**
