# Supplementary material for: Changes in the proteomes of the hemocytes and fat bodies of the flesh fly Sarcophaga bullata larvae after infection by Escherichia coli
Source: Proteome Sci. 2010 Jan 13;8:1. doi: 10.1186/1477-5956-8-1 (PMC2817646; doi:10.1186/1477-5956-8-1)
Supplement: Additional file 2 — Trends in expression of proteins changed after immune challenge in S. bullata larvae hemocytes and fat bodies. The table is in the Microsoft word format (Table 2.doc). Spots are numbered the same as in Additional file 1 and Figure 1. For each spot we show the accession number to the Swiss-Prot database, the protein name, estimated relative quantity including standard error of the mean (n = 3) in induced/non-induced larvae (I/N) from the PDQuest output and the fold change at three points of time; 30 min., 6 hours and 22 hours post-injection. [file 1477-5956-8-1-S2.DOC]

**Table 2**

**Trends in expression of proteins changed after immune challenge in *S. bullata* larvae hemocytes and fat bodies.**

| Spot no. | Access. no. | Protein name (function) | 30min  Relat. spot quantity±SEM  I/N | Fold change  I/N | 6 hours  Relat. spot quantity±SEM  I/N | Fold change  I/N | 22 hours  Relat. spot quantity±SEM  I/N | Fold change  I/N |
| --- | --- | --- | --- | --- | --- | --- | --- | --- |
| **Proteins changed in hemocytes after immune challenge** | | | | | | | | |
| 36 | P45594 | **Cofilin/actin-depolymer. factor** | nd | x | 1491±207/  508±50 | 2.9 | 3879±402/  320±2.6 | 12.0 |
| ^a^37 | P14130 | **40S ribosomal protein S14** | 235±65/  181±52 | 1.3 | 2857±257/  991±97 | 2.9 | 901±32/  153±74 | 5.9 |
| 38 | Q1HQ70 | Transgelin | nd | x | 1134±301/  154±42 | 7.3 | 1876±541/  175±30 | 10.0 |
| 39 | P05047 | **Lectin subunit α** | 493±47/  182±41 | 2.7 | 921±42/  442±81 | 2.0 | 248±28/  84±35 | 2.9 |
| 40 | P12613 | **T-complex protein 1 sub. α** | 809±49/  506±44 | 1.6 | 89±23/  758±97 | 0.1 | 40±2.6/  455±183 | 0.1 |
| 41 | P12613 | **T-complex protein 1 sub. α** | 840±100/  599±186 | 1.4 | 35±8.6/  306±40 | 0.1 | nd | x |
| ^b^42 | Q9Y0B4  Q94511 | **Prophenoloxidase 1**  **NADH-ubiquinone oxidoreductase sub.** | 194±9/  41±8 | 4.7 | 800±85/  50±17 | 16.0 | 30±12/  13±0.9 | 2.3 |
| **Proteins changed in fat bodies after immune challenge** | | | | | | | | |
| 43 | P42860 | **Glutathione S-transferase 1-1** | 1729±472/  777±148 | 2.2 | 1450±154/  917±368 | 1.6 | 755±38/  3222±147 | 0.2 |
| 44 | B4M6F3 | **GJ10765 (ferritin-like)** | 990±197/  397±76 | 2.5 | 830±87/  865±208 | 1 | 815±93/  1661±222 | 0.5 |
| 45 | Q9NDP1 | **Anterior fat body protein** | 2044±169/  925±121 | 2.2 | 467±141/  985±196 | 0.5 | 188±5/  846±88 | 0.2 |
| ^c^46 |  | unidentified | nd | x | 292±50/  91±38 | 3.2 | 326±5.5/  15±7 | 21 |

Spots are numbered the same as in Table 1 and Figure 1. For each spot we show the accession number to the Swiss-Prot database, the protein name, estimated relative quantity including standard error of the mean (n=3) in induced/ non-induced larvae (I/N) from the PDQuest output and the fold change at three points of time; 30 min., 6 hours and 22 hours post-injection.

**a) Measured and theoretical values of pI differ inadequately. The identification was not reliable.**

**b) Prophenoloxidase was identified in the spot from induced hemocytes. NADH-ubiquinone oxidoreductase was identified from non-induced hemocytes.**

**c) The spot has the same localization in the 2-D protein map of the fat bodies as the spot 38 (transgelin) identified from the hemocytes.**
